# Intrinsic and membrane-facilitated α-synuclein oligomerization revealed by label-free detection through solid-state nanopores

**DOI:** 10.1038/srep20776

**Published:** 2016-02-11

**Authors:** Rui Hu, Jiajie Diao, Ji Li, Zhipeng Tang, Xiaoqing Li, Jeremy Leitz, Jiangang Long, Jiankang Liu, Dapeng Yu, Qing Zhao

**Affiliations:** 1 State Key Laboratory for Mesoscopic Physics, School of Physics, Peking University, Beijing 100871, People’s Republic of China; 2 Collaborative Innovation Center of Quantum Matter, 100084 Beijing, China; 3 Center for Mitochondrial Biology and Medicine, The Key Laboratory of Biomedical Information Engineering of Ministry of Education, School of Life Science and Technology and Frontier Institute of Life Science, Frontier Institute of Science and Technology (FIST), Xi’an Jiaotong University, Xi’an 710049, China; 4 Department of Molecular and Cellular Physiology, and Howard Hughes Medical Institute, Stanford University, Stanford, California 94305, USA

## Abstract

α-Synuclein (α-Syn) is an abundant cytosolic protein involved in the release of neurotransmitters in presynaptic terminal and its aberrant aggregation is found to be associated with Parkinson’s disease. Recent study suggests that the oligomers formed at the initial oligomerization stage may be the root cause of cytotoxicity. While characterizing this stage is challenging due to the inherent difficulties in studying heterogeneous and transient systems by conventional biochemical technology. Here we use solid-state nanopores to study the time-dependent kinetics of α-Syn oligomerization through a label-free and single molecule approach. A tween 20 coating method is developed to inhibit non-specific adsorption between α-Syn and nanopore surface to ensure successful and continuous detection of α-Syn translocation. We identify four types of oligomers formed in oligomerization stage and find an underlying consumption mechanism that the formation of large oligomers consumes small oligomers. Furthermore, the effect of lipid membrane on oligomerization of α-Syn is also investigated and the results show that 1,2-dioleoyl-sn-glycero-3-[phospho-L-serine] (DOPS) small unilamellar vesicles (SUVs) dramatically enhances the aggregation rate of α-Syn while do not alter the aggregation pathway.

α-Synuclein (α-Syn) is a small intrinsically disordered cytosolic protein of 140 amino acids (14.3 kDa) that is expressed abundantly in human brain tissue. α-Syn has been reported to function in the control of synaptic vesicle fusion and thus the release of neurotransmitters, subsequent vesicle recycling, and maintenance of synaptic integrity^1^,^2^,^3^,^4^. However, in pathophysiological conditions, adequate α-Syn monomers in solution undergo a nucleation-dependent mechanism growing into oligomers, which are able to elongate to form profibers through monomers addition and finally mature fibers that form the hallmark subcellular structure Lewy bodies^5^,^6^,^7^. It is well acknowledged that the abnormal aggregation process of α-Syn monomers is associated with pathophysiology of Parkinson’s disease (PD) and dementia^7^,^8^,^9^. Recent studies have shown that initial α-Syn oligomerization may be the root cause of cytotoxicity rather than the fibers themselves. Conway *et al.* distinguished sphere-like oligomers of different sizes in addition to chain-like and ring-like profibers during α-Syn aggregation by atomic force microscopy (AFM)^10^,^11^,^12^. Lashuel *et al.* identified pore-like oligomers in the aggregation of α-Syn mutations using negative stain transmission electron microscopy (TEM), which is suggested to be the cause of cell dysfunction and even cell death in PD^13^,^14^.

Natively, α-Syn localizes to synaptic vesicles and appears both in soluble and membrane-associated forms^15^,^16^. The α-Syn-lipid membrane interaction is supposed to be responsible for triggering the normal function of α-Syn^5^. However, considerable researches postulate that the lipid vesicles may both accelerate and inhibit α-Syn fibril formation depending on the varying experimental conditions^6^,^17^,^18^. Zhu *et al.* investigated the effects of lipid vesicles on the conformation and fibrillation kinetics of α-Syn through Thioflavin T (ThT) fluorescence assays, circular dichroism (CD) measurements, and AFM, suggesting that the lipid membrane of different composition modulates the α-Syn aggregation process in different ways^19^,^20^. Recently, Hellstrand *et al.* revealed that the lipid membrane could co-aggregate with α-Syn fibers which can influence the structure and function of lipid membrane and α-Syn-formed amyloid deposit by using phospholipid quantification, polarization transfer solid-state nuclear magnetic resonance and cryo-TEM^21^. In 2015, Galvagnion *et al.* demonstrated that the presence of anionic lipid 1,2-dimyristoyl-*sn*-glycero-3-phospho-L-serine (DMPS) small unilamellar vesicles (SUVs) dramatically accelerated the α-Syn primary nucleation process by ThT fluorescence assays, resulting in three orders or more of magnitude enhancement in overall aggregation rate^22^. These studies provide a deeper understanding of the effect of lipids on the kinetics and overall process of α-Syn aggregation.

However, identifying different oligomer types and tracking their oligomerization dynamics is difficult due to the inherent challenges of studying heterogeneous and transient system by using traditional biochemical techniques. Imaging techniques, such as TEM and AFM, are capable of characterizing morphologies of α-Syn aggregates, but the results will inevitably be affected by the biased adsorption of aggregates to sample substrates^12^,^13^,^14^,^23^,^24^,^25^. Circular dichroism (CD), Fourier transform infrared spectroscopy, fluorescence and Raman spectroscopy can reveal the ensemble structure information, while have difficulties in identifying oligomer species. Light-scattering techniques, which permit *in situ* measurements, are not suitable due to the poor performance for heterogeneous systems. Recently, Cremades *et al.* adapted improved single molecule two color coincidence detection (smTCCD) and intermolecular fluorescence resonance energy transfer (FRET) technique to investigate this heterogeneous and transient system, and successfully identified two type of oligomers, the normal form and the toxic form^26^. However, these experiments required fluorescence labeling paradigms.

A nanopore sensor is a device based on Coulter Counter^27^: a nanometer-scale pore imbedded in a thin membrane separating two electrolyte-filled reservoirs. Driven by electric force, a charged analyte suspended in solution translocates through the nanopore, producing an ionic current drop due to the displacement of electrolyte volume in the nanopore. The statistical analysis of the ionic current drops and their duration time can reveal the geometry and charge properties of the analyte^28^. Nanopore technology was proposed for the ultimate goal of high-throughput DNA sequencing nearly two decades ago^29^,^30^. And now it has been widely extended to detect and investigate protein molecules, such as mapping the structural dimension^31^,^32^,^33^,^34^, investigating the chemical bond^35^, describing DNA-complex interaction^34^,^36^, differentiating folded and unfolded protein state^37^,^38^,^39^,^40^, and distinguishing different microRNAs^41^. There are also a few reports on detection of α-Syn using protein nanopores (α-hemolysin)^42^,^43^ and it has been reported that the biological nanopore could offer the earliest steps of the α-synuclein aggregation pathway and provides the potential basis for the development of drugs that can prevent α-Syn aggregation at the initial stage. Solid-state nanopores, with a fabricated pore drilled in a thin insulating membrane, have advantages in controllable fabrication, tunable nanopore size and geometry, and long stability in aqueous solution^44^,^45^,^46^,^47^,^48^,^49^,^50^. As an *in situ* and label-free single molecule detection method with fairly high resolution, solid-state nanopores provide a viable and unique approach to characterize the oligomers formed during the process of aberrant protein aggregation, such as Amyloid-β protein associated with the Parkinson’s disease^51^ and lysozyme^52^.

In this study, we use solid-state nanopores to study the oligomerization of α-Syn protein in a label-free and single-molecule approach. A tween 20 surface coating method was developed to enable continuous and smooth α-Syn translocation through SiN nanopores. Through analysis of translocation events for α-Syn incubated over a range of times, we identified four types of oligomers formed during aggregation under controlled incubation condition. These four types of oligomers inferred as intermediates show a time-dependent quantity fraction change, suggesting that the formation of large oligomers is based on the consumption of small oligomers. Furthermore, we investigated the effect of lipid SUVs on α-Syn oligomerization process. The experimental results indicate that it is the presence of 20% 1,2-dioleoyl-sn-glycero-3-[phospho-L-serine] (DOPS) dramatically enhances the aggregation rate of α-Syn, but does not alter the aggregation pathway. Together, our data describes an effective label-free single-molecule methodology that permits the detailed investigation the oligomerization stage of α-Syn protein both in isolation and in the presence of synthetic vesicles that mimic *in situ* conditions.

## Results

### α-Syn protein translocation through Tween 20-coated nanopore

**Figure 1:**
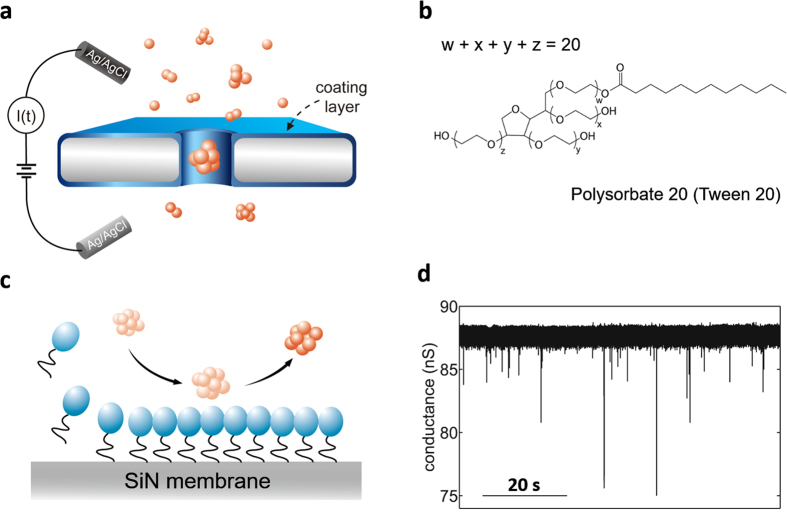
Detection of α-Syn using tween 20 coated solid-state nanopore. **(a)** Schematic diagram of experimental setup. The flow cell is separated by silicon nitride membrane with a nanopore embedded on it. In order to provide a non-specific adsorption surface, the silicon nitride membrane is coated by a layer of tween 20 molecules (light blue layer). The α-Syn monomer is represented by particles just for clear reading in schematic diagram, which may not reveal its real morphology. **(b)** The chemical structure of tween 20. **(c)** Illustration of the assemble process of tween 20 on hydrophobic silicon nitride membrane and the compact coating layer reduce irreversible non-specific adsorption of α-Syn oligomers. Light blue ellipsoids and solid black lines represent hydrophilic ethylene glycol head groups and the hydrophobic alkyl tail of tween 20 molecule, respectively. **(d)** Current traces of nanopore experiment using tween 20 coated nanopore under 100 mV. The α-Syn sample had been incubated for 96 h at pH 9.

Figure 1a shows the schematic diagram of α-Syn protein detection experimental setup. A flow cell is separated by a silicon nitride membrane (Fig. 1a gray substrate) of around 40 nm in effective thickness, in which a 20 nm nanopore was drilled using focused electron beam in TEM. Two Ag/AgCl electrodes connected to patch-clamp amplifier were immersed in two reservoirs of the flow cell filled with electrolyte solution (1 M KCl, 10 mM HEPES, pH 9) to apply electric field across nanopore and to record the current signal through the nanopore. α-Syn protein samples (diluted to 1 μg/ml, 0.69 μM) after incubation under well-controlled condition were added into the cis side of the flow cell. An electric field was then applied across the nanopore to drive translocation of α-Syn proteins, leading to characteristic current blockade on the ionic current trace.

We found that bare nanopore is easily and irreversibly clogged by protein molecule adsorption on the nanopore walls by observing a dramatic current drop and a subsequent fluctuating current trace (Supplementary Fig. 1). It is believed that this phenomenon is caused by irreversible adsorption of α-Syn inside nanopore due to strong interaction between proteins and silicon nitride nanopore^53^,^54^. The non-specific adsorption is a common phenomenon^51^,^52^,^55^ and have been one of challenges impeding the successfully compliment of solid-state nanopore experiment. Several groups have made useful attempts to solve this problem, such as lipid coating^51^,^56^ and using glass nanopore^52^ to reduce the non-specific adsorption of aggregation-prone protein, PEG coating^57^,^58^,^59^ and chemically modified solid-state nanopore^60^ to inhibit the adsorption of DNA and other protein complex. Here by surface coating a layer of tween 20 molecules onto nanopore, we achieved smooth and continuous current trace with successful translocation of α-Syn samples (Fig. 1b–d). Tween 20 is a commercial used non-ionic surfactant (molecular weight: 1227.54 g/mol) consisting of hydrophilic ethylene glycol head groups and a hydrophobic alkyl tail (Fig. 1b). Here by self-assembling a compact layer of tween 20 on hydrophobic SiN surface (Supplementary Fig. 2), with its hydrophilic head groups exposed to solution and hydrophobic tail contacting with the hydrophobic SiN surface (Fig. 1c), irreversible non-specific adsorption of α-Syn proteins has been significantly reduced^51^,^61^,^62^,^63^,^64^, as evidenced by fluorescence microscopy experiments (Supplementary Fig. 3). The smooth current traces recorded from tween 20 coated nanopore (Fig. 1d) enables continuous translocation of α-Syn samples. Tween 20 exhibits an excellent ability against protein adsorption without affecting the protein’s original structure^61^,^62^,^63^,^64^, which is preferred over other lipid coating methods, since α-Syn-lipid membrane interaction have been implicated in affecting protein and membrane properties^5^,^6^,^20^,^22^,^65^.

### Identifying four types of oligomers in α-Syn samples

**Figure 2:**
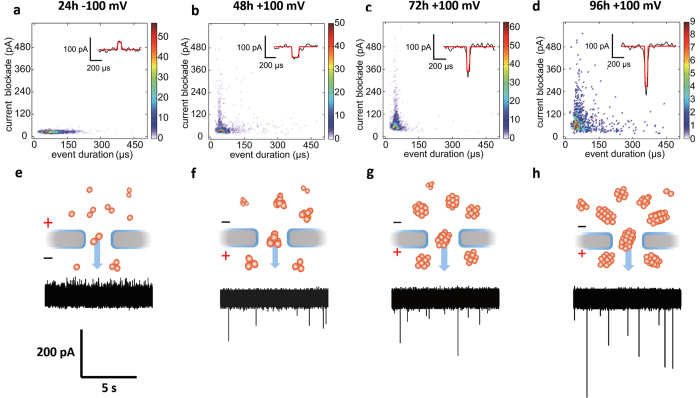
Translocation events of α-Syn samples incubated for different time. Density histograms for translocation events of α-Syn only samples incubated for **(a)** 24 h, **(b)** 48 h, **(c)** 72 h and **(d)** 96 h. The insets are typical events for different α-Syn only samples. The black lines in these insets are filtered current trace and the red lines are the fitted square waves. Schematic graphs and characteristic current trace are also presented for **(e)** 24 h, **(f)** 48 h, **(g)** 72 h and **(h)** 96 h samples. Small orange spheres present α-Syn monomers. Under controlled incubation condition, α-Syn monomers begin to aggregate and grow bigger with longer incubation time, leading to deeper translocation events.

α-Syn samples were incubated for 24 h, 48 h, 72 h, and 96 h under well-controlled conditions prior to addition to the cis side of the flow cell. A temperature-controlled shaker (200 rpm, 37 ^o^C) was used to accelerate the aggregation. Fig. 2a–d shows the density histograms for translocation events of α-Syn samples that had been incubated for 24 h, 48 h, 72 h, and 96 h, respectively. The insets are the corresponding typical current traces of single translocation events. Every translocation event is characterized by two significant parameters, current blockade ΔI and event duration Δt, corresponding to the exclude volume (i.e. size) and dwell time of proteins inside the nanopore. Fig. 2e–h depicts the scheme of the oligomerization of α-Syn and its corresponding original experimental current traces recorded for α-Syn samples incubated for different times. α-Syn monomers aggregate into oligomers and grow bigger along with longer incubation time, showing larger current blockades. Note that −100 mV instead of +100 mV is applied for 24 h sample to see successful translocation events due to the domination of electroosmosis as the driving force for α-Syn translocation in 24 h sample (Supplementary Fig. 4)^66^.

**Figure 3:**
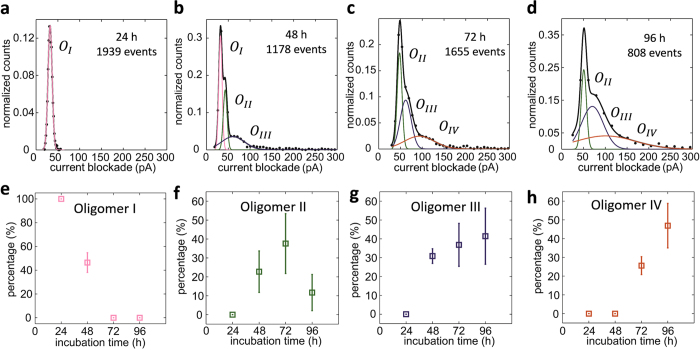
Multi-Gaussian fitting results of current blockade histograms for α-Syn only samples. Current blockade histograms and multi-Gaussian fitting results for **(a)** 24 h, **(b)** 48 h, **(c)** 72 h and **(d)** 96 h α-Syn only samples. The blockade histograms are normalized to the number of total events in each sample for clarity. The black solid lines are the Gaussian fitting results carried on the original data marked by black dot. The value of 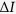 is cut off at 300 pA for statistical analysis. The individual Gaussian components are classified into four types, *O*_*I*_, *O*_*II*_, *O*_*III*_, and *O*_*IV*_, based on its peak position. *O*_*I*_, *O*_*II*_, *O*_*III*_, and *O*_*IV*_ are marked by pink, green, purple and orange solid lines, respectively. The percentage changes of under-curve area for each Gauss contributions over incubation time for **(e)** oligomer I, **(f)** oligomer II, **(g)** oligomer III and (**h**) oligomer IV are depicted. The error arises from fitting error.

Figure 2a–d shows an increase in current blockade as a function of incubation time with increasing value of mean current blockade <ΔI>, from 32.1 pA (24 h) to 35.6 pA (48 h), to 53.0 pA (72 h), to 64.3 pA (96 h), indicating the aggregation nature of α-Syn^8^,^9^. Subsequently, multi peak Gaussian fitting is used to probe more details in current blockade distribution for 24 h, 48 h, 72 h, and 96 h samples and the fitting results are shown in Fig. 3a–d. Multi-Gaussian fitting was required to fit these distribution curves to give flat residuals except for 24 h sample (fitted by only one Gaussian component). Surprisingly, the peaks of Gaussian components from four samples can be well cataloged into four populations, O_I_ to O_IV_ based on the position of the peaks within a small error bar range. Detailed fitted peak positions for the four populations are summarized in Table 1. For instance, for the O_III_ peak, it positions at 61.0 pA, 60.7 pA and 65.4 pA in 48 h, 72 h, and 96 h samples, demonstrating a well-defined behavior. It is noteworthy to state that we did not observe translocation signal for non-incubated sample referred as 0 h sample which contains almost exclusively monomers and due to the small size of monomers (14.3 kDa) it is likely that their translocation events are below the resolution limit in our experiments. Therefore, we classify the observed populations in Fig. 3a–d into four types of oligomers: O_I_, O_II_, O_III_, and O_IV_ since mature fibers are much bigger than the nanopore size and they cannot be detected in our system.

Thanks to the advantages of label-free and single molecule detection based on nanopore experiments, we are able to probe distinct species within heterogeneous system based on the current blockade distribution of translocation events. The sample incubated under the aggregation promoting conditions for 24 h consists of almost only small oligomers (O_I_) of similar size based on its uniform and narrow distribution of current blockades (Fig. 3a), confirmed by negative stain TEM results (Supplementary Fig. 5). The peak position of Gaussian components listed in Table 1 reveal that distinct species of oligomers appeared after 48 h incubation (Fig. 3b–d), also supported by TEM analysis (Supplementary Fig. 5), presenting a heterogeneous system involving oligomers with different sizes in 48 h, 72 h, 96 h samples.

According to previous studies in protein translocation through nanopores^28^,^33^,^37^,^67^, for the analyte that is much smaller than an idealized cylindrical nanopore, the instantaneous excluded volume 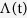 of the analyte is approximately proportional to 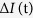 and the relationship is expressible as follows:


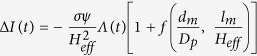


where 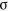 is the solution conductivity, 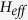 is the effective length of solid-state nanopore, and 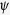 is the applied voltage. The correction factor 
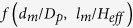
 depends primarily on the geometry of nanopore and analyte. For an analyte much smaller than the length of nanopore, the correction factor 
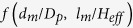
 is approaching zero and the exclude volume of the analyte can be estimated from 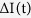 with 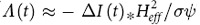
67. An effective length of 40 nm was used here to calculate the exclude volume and the diameter
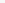
of the defined four types of α-Syn oligomers. The detailed results are listed in Table 1. The calculated diameters for the four fitted clusters are 4.4 nm, 5.0 nm, 5.5 nm, and 6.5 nm. The general agreement between calculated diameter and that reported by other groups characterized by AFM demonstrate the rationality of this method^5^,^12^.

The percentage of under-curve area for each Gaussian component is proportional to the number of captured molecules. Therefore, we are able to monitor the time-dependent quantity fraction of the four types of oligomers and give kinetic information of α-Syn oligomerization. The percentage of total under-curve area for each Gaussian components in 24 h, 48 h, 72 h, and 96 h samples have been calculated and the data is illustrated as a function of incubation time in Fig. 3e–h. The percentage for the smallest oligomer O_I_ gradually decreased after 24 h (Fig. 3e), indicating its consumption during the aggregation of α-Syn. The percentage for O_II_ increases from 48 h to 72 h and decreases after 96 h incubation (Fig. 3f). O_III_ oligomers appear at 48 h and grow steadily in percentage from 48 h to 96 h (Fig. 3g). O_IV_ oligomers emerge at 72 h and exhibit a sharp increase in percentage at 96 h (Fig. 3h). From above analysis, it is reasonable to assure that at least four types of α-Syn oligomers have formed during the aggregation process under well-controlled condition and these four types of oligomers undergo a conversion between one another. In the 24 h sample, O_I_ oligomers form and serve as aggregation seeds. As incubation time increases, oligomers O_II_, O_III_, and O_IV_ appear by the consumption of O_I_, resulting in reduction of quantity fraction of O_I_. The quantity fraction of O_II_ reaches its maximum after 72 h incubation and then declines maybe because they are precursors for O_III_ and O_IV_. Both of the quantity fraction of oligomers O_III_ and O_IV_ increase with longer incubation (up to 96 h), but they show different behavior indicating they are two distinct species. According to Smoluchowski rate equation, the rate of proteins captured by nanopore (J) can be expressed as: 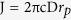. In this equation, c is the bulk concentration of molecules; D is the bulk diffusion constant, and 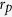 is the capture radius of nanopore, which is determined by the geometric parameters of nanopore and the applied voltage. Although the 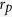 can be regarded as a constant value approximately in 48 h, 72 h, and 96 h samples for the same voltage applied and similar geometry of nanopore used in these experiment, the diffusion constant of different types of oligomer with dissimilar sizes are not the same, leading to an underestimation of percentage for larger oligomers with smaller diffusion constant in sample^51^.

The current blockade histograms for various samples showed heterogeneous distribution, which were attributed to multiple oligomers formation during incubation. The event duration histograms for different samples (Supplementary Fig. 6) do not exhibit the same characteristic of heterogeneity. The first passage theory was used to fit the duration histograms and the extracted values of drift velocity, v. From four samples, we found that drift velocity increases along with incubation time up to 72 h, implying a faster translocation speed of samples with longer incubation time due to an increasing charge per aggregate from addition of monomers^51^.

### Investigation of the effect of lipid SUVs on the kinetics of α-Syn’s oligomerization

**Figure 4:**
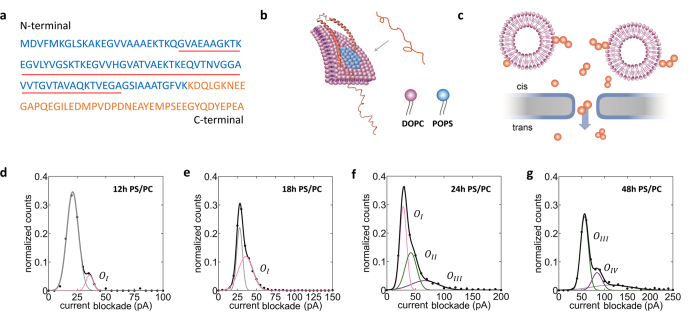
Detection of α-Syn and PS/PC SUVs co-incubated samples. **(a)** Protein sequence of wild type α-Syn. The positive charged N-terminal (residues 1–106) of α-Syn is colored blue. The C-terminal is labeled by orange color and the NAC region is denoted by red underline. **(b)** Illustrating graph that shows the transition of an unstructured α-Syn molecule into an α-helices structure upon binding to lipid membrane. The N-terminal adopts the α-helices structure and acts as an anchor when α-Syn binds to phospholipid and the C-terminal is usually unstructured. **(c)** Schematic diagram of nanopore experiment for α-Syn and lipid SUVs co-incubated sample. Current blockade histograms for PS/PC co-incubated samples for **(d)** 12 h, **(e)** 18 h, **(f)** 24 h and **(g)** 48 h are shown. The original data is marked by black dot; multi-Gaussian fitted curves are labeled by black solid line. Individual Gaussian components are classified depending on oligomeric species defined in α-Syn only samples and marked by corresponding color. The error is from fitted error.

The presence of lipid membranes is believed to have a fundamental influence on the kinetics of α-Syn oligomerization, which depends on the relative proportion of the protein and lipids, as well as the lipid compositions used^6^. SUVs used here are composed of 20% 1,2-dioleoyl-sn- glycero-3- [phospho-L-serine] (DOPS) and 80% 1-palmitoyl-2-oleoyl-sn-glycero-3-phosphocholine (POPC) to imitate the lipid constitution of native vesicles. We investigated the effect of composite phospholipids SUVs consisting of 20% DOPS and 80% POPC (PS/PC) on the oligomerization of α-Syn. Samples were incubated under 37 °C in quiescence conditions to provide full interaction between PS/PC and α-Syn proteins. Figure 4a presents the amino acid sequence of α-Syn protein, which can be classified into three domains: a) the positive charged N-terminal that contains highly conserved and imperfect repeats KTKEGV, residues 1–60; b) the hydrophobic core at the central region, which is known as the non-amyloid-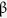 component (NAC), residues 61–90; c) an acidic C-terminal, residues 96–140. Although intrinsically unfolded in solution, the N-terminal of α-Syn will adopt an α-helix structure upon binding to lipid membrane, while the acidic C-terminal remains unfolded, as shown in Fig. 4b5^68^,^69^.

Figure 4c shows the schematic diagram of nanopore experiment for PS/PC and α-Syn co-incubated samples. The size of PS/PC SUVs is around 60 nm (Supplementary Fig. 7a), which is much larger than the 20 nm nanopore used here. Prior to addition of sample, ultrasonic treatment is adopted to break the weak binding between α-Syn and lipid membrane. This ultrasonic treatment is found to have little effect on α-Syn aggregates (see Supplementary Fig. 8 for details). It is believed that the translocation events belong to the α-Syn oligomers other than the α-Syn-SUVs complex for its similar behavior of blockade histograms as samples without SUVs.

Current blockade histograms for lipid co-incubated α-Syn samples incubated for 12 h, 18 h, 24 h, and 48 h under quiescence condition are presented in Fig. 4d–g. Multiple Gaussian fitting is used to fit the multi-peak histograms. The range that the current blockade values cover becomes wider and multiple peaks emerge as the increase of incubation time. According to the fitted peak values (Supplementary Table 1), the peaks can also be categorized into four types of oligomers as we previously defined in α-Syn translocation experiment. The O_I_ Gaussian component appeared in 12 h, 18 h and 24 h PS/PC samples and shows an increase in percentage of under-curve area along with longer incubation time, indicating more O_I_ oligomers has formed as a function of incubation time. As time increases, the defined larger oligomers such as O_II,_ O_III_ and O_IV_ have emerged indicating the aggregation nature of lipid co-incubated α-Syn samples. The analogous evolution behavior of defined oligomeric types in lipid co-incubation samples compared with α-Syn only samples suggests that the formation of larger oligomers is also based on the consumption of small oligomers formed earlier.

**Figure 5:**
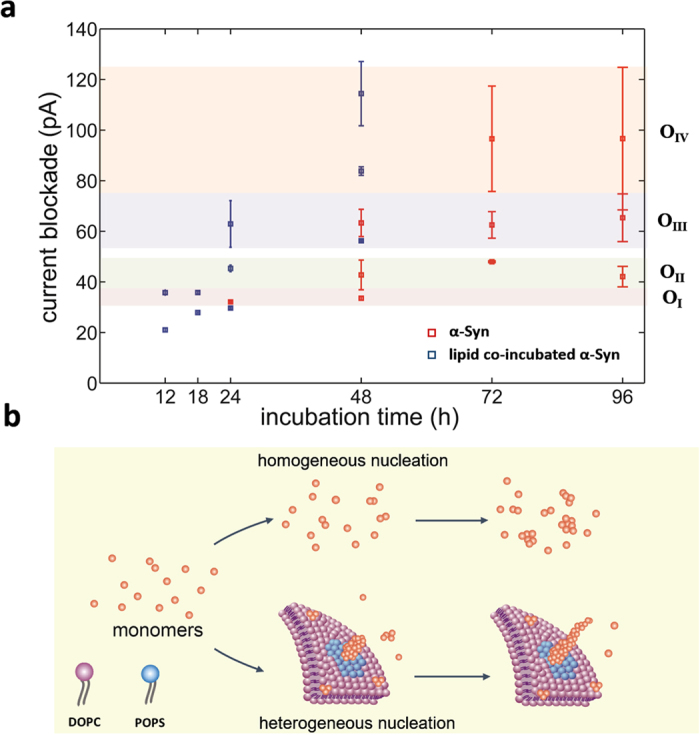
**Comparison between α-Syn only and PS/PC SUVs co-incubated α-Syn samples**. **(a)** Multi-Gaussian fitted peak values of α-Syn only sample (marked by red square) and PS/PC co-incubation α-Syn sample (marked by blue square) in current blockade distributions as a function of incubation time. The pink, green, purple, and orange bands indicate four types of oligomers we discussed above. The band width is determined by error bar. (b) The scheme depicts the possible mechanism of α-Syn aggregation process in the absence or presence of PS/PC SUV. Note only part of the lipid SUVs is shown in the scheme to show the binding area more clearly.

In order to give an intuitive comparison of the oligomeric species formed in α-Syn only sample and PS/PC SUVs co-incubated α-Syn sample, the multi-Gaussian fitted peak values are plotted as the function of incubation time in Fig. 5a. It can be seen that the peak values of current blockade for PS/PC SUVs co-incubated α-Syn sample fall into an acceptable region of the corresponding four oligomers defined in α-Syn only samples although the kinetics is accelerated. For α-Syn only sample, 24 hours incubation only results in small oligomer O_I_; after 48 hours incubation, larger oligomer O_II_ and oligomer O_III_ appear. For samples incubated for more than 72 hours, small oligomer O_I_ disappears and large oligomer O_IV_ emerges. For PS/PC SUVs co-incubated α-Syn sample, the aggregation pathway is similar to that of α-Syn only sample, which also goes through the conversion of four types of oligomer, while the time need for the formation of large oligomer is reduced from 96 h to 48 h, leading to an 100% enhancing in aggregation rate. Note that the incubation condition for these two samples is not exactly the same: we used an aggregation-promoting condition for the α-Syn only sample to speed up the experiment schedule and a quiescence condition for SUVs co-incubated sample to guarantee full interaction between SUVs and α-Syn, therefore the aggregation rate enhancement is underestimated here. In addition, two small peaks (<30 pA) have been observed lipid SUVs co-incubated α-Syn samples under 12 h and 18 h incubation, which may be due to folded state of α-Syn when binding to lipid membrane.

It is acknowledged that the anionic lipid DOPS SUVs are responsible for this enhancement of aggregation rate. The possible mechanism is that the negative charged DOPS attracts positive charged N-terminal of α-Syn, leading to a locally high concentration of α-Syn monomers, which accelerates the primary nucleation process^22^,^65^. We also did the control experiment with 100% POPC SUVs co-incubated α-Syn samples incubated for 6 h, 12 h, 24 h, and 48 h (Supplementary Fig. 9). According to multi-Gaussian fitting results, no peak larger than 60 pA can be seen in current blockade histograms after 48 hours incubation, indicating a very slow aggregation rate, which agrees with results reported by other groups^65^. Control experiments suggest that POPC has no notable effect in α-Syn aggregation, and the enhanced aggregation rate is mainly caused by DOPS in lipid SUVs.

Figure 5b illustrates a possible mechanism of α-Syn aggregation process in the absence or presence of lipid SUVs. The aggregation process of α-Syn protein exhibits a sigmoidal growth profile, which has an initial lag phase due to the high barrier of nuclei formation^26^. In the absence of PS/PC SUVs, the α-Syn monomers form nuclei through a high barrier homogenous nucleation process, leading to a relatively slow aggregation rate (Fig. 5b). While in the presence of PS/PC SUVs, the α-Syn monomers form nuclei through a low barrier heterogeneous nucleation process^68^. In addition, the weak attractive force between the negatively charged DOPS hydrophobic head and the positive α-Syn N-terminal attracts more monomers to gather around DOPS molecules, leading to a local relatively higher concentration of α-Syn monomers, which contributes to the growth of nuclei to large oligomers^65^. Both of these two factors count for the enhancement of aggregation rate for the PS/PC SUVs co-incubated α-Syn sample. Our experimental results indicate the presence of PS/PC SUVs only accelerates the aggregation rate of α-Syn while does not alter the aggregation pathway.

## Discussion

In this study, we have demonstrated that solid-state nanopore sensors can investigate the time-dependent intrinsic and membrane-facilitated oligomerization of α-Syn in a single-molecule and label-free approach. Tween 20 coating is found to effectively inhibit non-specific adsorption of α-Syn on silicon nitride nanopore membrane. Four types of oligomers were identified as intermediates formed during α-Syn aggregation by multi-Gaussian fitting of current blockade distributions. The quantity fraction of these oligomers is found to change over time, suggesting a consumption mechanism of small oligomers for the formation of large oligomers. In addition, we found that the negative charged DOPS dramatically accelerate the aggregation rate of α-Syn and the PS/PC SUVs co-incubated samples share similar α-Syn aggregation pathway with the α-Syn only sample. As a label-free single molecule detection method, solid-state nanopores are suitable for characterizing heterogeneous and vital oligomerization of α-Syn *in situ*, giving important insights into the formation and conversion of oligomeric species, which not only represent significant steps of aggregation process but also regarded as key role in the pathogenesis of PD.

## Method

### Nanopore Fabrication

2 μm-thick silicon oxide film and a layer of 200 nm thick silicon nitride were deposited on both sides of the 400 μm-thick silicon wafers. Then photolithography and reactive ion etching (RIE) were used to form a square of 20 μm × 20 μm freestanding membrane of two layers (SiN/SiO_2_). A focused ion beam (FIB, DB235) was adopted to remove about 1.5 μm thick silicon oxide of 1 μm × 1 μm in the center of the freestanding membrane, and the remaining 500 nm thick silicon oxide was removed by a subsequent timed buffered oxide etch (BOE), resulting in a 2 μm × 2 μm freestanding mini membrane of silicon nitride. The outside silicon nitride layer was estimated to be 120 nm by ellipsometry after the timed hot KOH thinning process. The chips were cleaned with NH_4_OH, H_2_O_2_ and water (1:1:6 v/v) right after RCA. Then the chips are dried by nitride-flow before nanopore drilling. The nanopores were drilled in the center of mini membranes by a 300 kV focused electron beam from transmission electron microscope (TEM, Tecnai F30). Note the effective thickness of SiN membrane is ~40 nm due to the truncated double-cone geometry of the drilled pore^70^,^71^. We store the drilled chips in air in a constant temperature and humidity drying oven for further use.

### Tween 20 Coating Buffer Preparation

0.1% w/v Tween 20 was added in 1 M KCl with 10 mM HEPES buffer at pH 9. The mixture solution was stored at room temperature for further use.

### Contact Angle Experiment

20 chips are separated to two groups as bare SiN membrane and tween 20 coated ones to compare the difference of hydrophilicity. Tween 20 coated chips are bathed in 0.1% w/v tween 20 solution for 1 h at room temperature and blew-dry by nitrogen gas gun. Control uncoated chip samples are bathed in pure water for 1 h at room temperature. Contact angle experiments are conducted by dripping a drop of pure water onto the membrane surface, after which the profile is captured by the optical system of a contact angle goniometer (Datephysics OCA20). The contact angle between liquid/solid interface and liquid/vapor interface is then analyzed automatically by software.

### Fluorescent Experiment

Cover slip (Fisher Brand, Microscope cover glass: 12-545-E 22*60-1) is cleaned by solution (NH_4_OH : H_2_O_2_ : water = 1:1:6) at 85 °C for 20 min, then rinsed by ultrapure water and blew-dry by Nitrogen gas gun. After the cleaning process, PDMS micro flow channels of 40 μm in width and 10 μm in height is mounted on the cover glass. The dyed α-Syn sample diluted by the buffer with and without 0.1% w/v Tween 20 are loaded separately through the tiny hole connecting to the micro fluidic channel. The fluorescent images are recorded after 10 min. Green fluorescence of YOYO-1 stained α-Syn oligomers anchored onto the glass surface is observed with Leica TCS SPE Confocal Microscope (Leica, German) after excitation with a 488 nm laser. Images are captured in 512 pixels × 512 pixels.

### Lipid SUVs Preparation

Two populations of small unilamellar vesicles (SUVs) containing different ratio of (mol/mol) 1-palmitoyl-2-oleoyl-sn-glycero-3-phosphocholine (POPC) and 1,2-dioleoyl-sn- glycero-3- [phospho-L-serine] (DOPS) (Avanti Polar Lipids, Birmingham, AL) were mixed in a molar ratio of 100:0 and 80:20, respectively. The mixtures were dried in a vacuum to form films. The membrane films were re-suspended in 10 mM HEPES buffer at the pH 7.45 and stirred at 45 °C for 1 hour. The sonication (Ultra Sonics, 760, 3 × 5 min, 30% maximum power) was used to prepare SUVs of the lipids.

### Dynamic Light Scattering Measurements

Dynamic light scattering measurements were carried out using a spectrometer of standard design (ALV-5000/E/WIN Multiple Tau Digital Correlator) with a Spectra-Physics 2017 200 mW Ar laser (wavelength: 514.5 nm). The scattering angle was 90 and CONTIN method was used to analyze the distribution of the radii of micelles. Unweighted data were recorded for all experiments. The scattering intensities were recorded and normalized with respect to the total concentration.

### α-Syn only Oligomers Preparation

The wild-type α-Syn proteins before aggregation was purchased from AnaSpec, Inc. in the form of powder. Then the powder was dissolved with 10 mM HEPES buffer at pH 7.45 to a concentration of 100 μg/ml (6.9 μm). For the sake of experiment, equimolecular concentrations of protein samples (6.9 μm) were sealed in 1 mL siliconized plastic microcentrifuge tube. For α-Syn only experiments, a tube containing 6.9 μm α-Syn at pH 7.45 was incubated under constant agitation at 200 rpm, 37 °C in the dark for 1–4 days to accelerate aggregation process. At each time point, 20 μl aliquot was diluted with Tween 20 buffer (10 mM HEPES, pH 9, 1 M KCl, 0.1% w/v Tween 20) into an experimental concentration of 0.69 μM α-Syn for nanopore translocation measurement.

### Lipid SUVs Doped α-Syn Oligomers Preparation

Wild-type α-Syn was prepared and diluted as above. Then 69 μm 80% PS/20% PC or 100% PC in the control experiment was added into the sample tube that means the molar ratio of α-Syn and lipid SUVs are 1:10, which will accelerate the aggregation process of α-Syn according to the work by Galvagnion *et al.*^22^. In order not to disrupt the interaction between α-Syn proteins and lipid SUVs surface, the samples are incubated at quiescence condition at the controlled temperature 37^o^C for 12 h, 18 h, 24 h, and 48 h in 10 mM HEPES buffer at pH 7.45. At each time point, 20 μl aliquot was diluted with Tween 20 buffer (10 mM HEPES, pH 9, 1 M KCl, 0.1% w/v Tween 20) into a concentration of 0.69 μM α-Syn and 6.9 μM lipid SUVs for nanopore translocation measurement.

### α-Syn Oligomers Translocation Experiments

The chips containing nanopore were mounted in a sealed fluid cell and were separated into two electrically isolated reservoirs of electrolytes. First the 0.1% w/v Tween 20 buffer was added into cis side and after 10 min the protein sample diluted with the 0.1% w/v Tween 20 was introduced into the cis side of the fluid cell. The voltage was applied through two Ag/AgCl electrodes coupled to the two opposite electrolyte reservoirs by an Axon 200B patch clamp amplifier (Molecular Devices, Sunnyvale, CA). Attached with an 8 pole, 40 kHz, low pass Bessel filter operating in resistive feedback mode, the patch clamp was also used for ionic currents measurement. The output of patch clamp was digitized at 250 kHz and continuously recorded by an Axon Digidata 1440A digitizer and pClamp 10.3 software. Then the data recorded was analyzed through custom MATLAB code (The MathWorks, Natick, MA). All the events showed in figures have been filtered by 10 kHz low-pass Bessel filter through MATLAB code for clarity.

### Samples for Transmission Electron Microscopy Preparation

TEM images were obtained by negative-staining method. First, 7 μl aliquots of 0.1 mg/mL α-Syn in 10 mM HEPES buffer at pH 7.4 were transferred to plasma processed, fresh glow-discharged, carbon-coated copper grids. After 1 minute, the sample solution was wicked off with a piece of filter paper and the grid was rinsed with fresh deionized water, then a 7 μl droplet of 2% (w/v) phosphotungstic acid staining solution was placed on the grid. After 1 minute, the excess fluid on the grid was wicked off and the grid was allowed to dry. The samples were viewed by a transmission electron microscope at 300 kV (TEM, Tecnai F30).

## Additional Information

**How to cite this article**: Hu, R. *et al.* Intrinsic and membrane-facilitated a-synuclein oligomerization revealed by label-free detection through solid-state nanopores. *Sci. Rep.* **6**, 20776; doi: 10.1038/srep20776 (2016).

## Supplementary Material

Supplementary Information

## Figures and Tables

**Table 1:** The multi-Gaussian fitted peak position 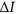, calculated excluded volume 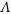, and calculated oligomer diameter 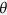 in 24 h, 48 h, 72 h, and 96 h α-Syn only samples.

Sample	24 h			48 h			units
cluster	*O*_*I*_	*O*_*II*_	*O*_*III*_	*O*_*I*_	*O*_*II*_	*O*_*III*_	–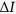	32.1 ± 2.2	–	–	34.2 ± 5.1	46.9 ± 7.7	61.0 ± 4.7	***pA***
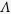	45.9 ± 3.1	–	–	48.8 ± 7.3	67.0 ± 11.0	87.1 ± 6.7	***nm***^3^
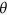	4.4 ± 0.1	–	–	4.53 ± 0.2	5.0 ± 0.3	5.5 ± 0.1	***nm***
**Sample**	**72 h**			**96 h**			**units**
**cluster**	***O***_***I**I*_	***O***_***III***_	***O***_***IV***_	***O***_***I**I*_	***O***_***III***_	***O***_***IV***_	**–**
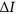	47.9 ± 2.4	60.7 ± 4.1	92.4 ± 20.3	43.2 ± 1.0	65.4 ± 4.7	98.3 ± 25.5	***pA***
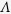	68.4 ± 3.4	86.7 ± 5.8	132.0 ± 29.0	61.7 ± 1.4	93.4 ± 6.7	140.4 ± 36.4	***nm***^3^
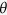	5.1 ± 0.1	5.5 ± 0.1	6.3 ± 0.5*	4.9 ± 0.1	5.6 ± 0.1	6.4 ± 0.5^*^	***nm***

The errors are fitting errors. The oligomers are regarded as spheres to calculate their diameter. (*the calculated diameter of O_IV_ based on the sphere model may lead to an overestimation of the actual size, which are more likely have an asymmetric short rod-like structure due to the broad distribution of blockage of O_IV_^56^ and the TEM image in Supplementary Fig. 5d.)
