# Supplementary material for: Intrinsic and membrane-facilitated α-synuclein oligomerization revealed by label-free detection through solid-state nanopores
Source: Sci Rep. 2016 Feb 11;6:20776. doi: 10.1038/srep20776 (PMC4749980; doi:10.1038/srep20776)
Supplement: Supplementary Information [file srep20776-s1.pdf]

Supplementary Materials for

Intrinsic and membrane-facilitated α-synuclein oligomerization revealed by label-free detection through solid-state nanopores

Rui Hu,[1,2] Jiajie Diao,*[3] Ji Li,[1,2] Zhipeng Tang,[1,2] Xiaoqing Li,[1,2] Jeremy Leitz,[4] Jiangang Long,[3] Jiankang Liu,[3] Dapeng Yu[1,2] Qing Zhao,*[1,2]

[1]State Key Laboratory for Mesoscopic Physics, School of Physics, Peking University, Beijing 100871, People's Republic of China.

[2]Collaborative Innovation Center of Quantum Matter, 100084 Beijing, China.

[3] Center for Mitochondrial Biology and Medicine, The Key Laboratory of Biomedical Information Engineering of Ministry of Education, School of Life Science and Technology and Frontier Institute of Life Science, Frontier Institute of Science and Technology (FIST), Xi'an Jiaotong University, Xi'an 710049, China.

[4]Department of Molecular and Cellular Physiology, and Howard Hughes Medical Institute, Stanford University, Stanford, California 94305, USA.

*Correspondence to: diaojj@gmail.com; zhaoqing@pku.edu.cn

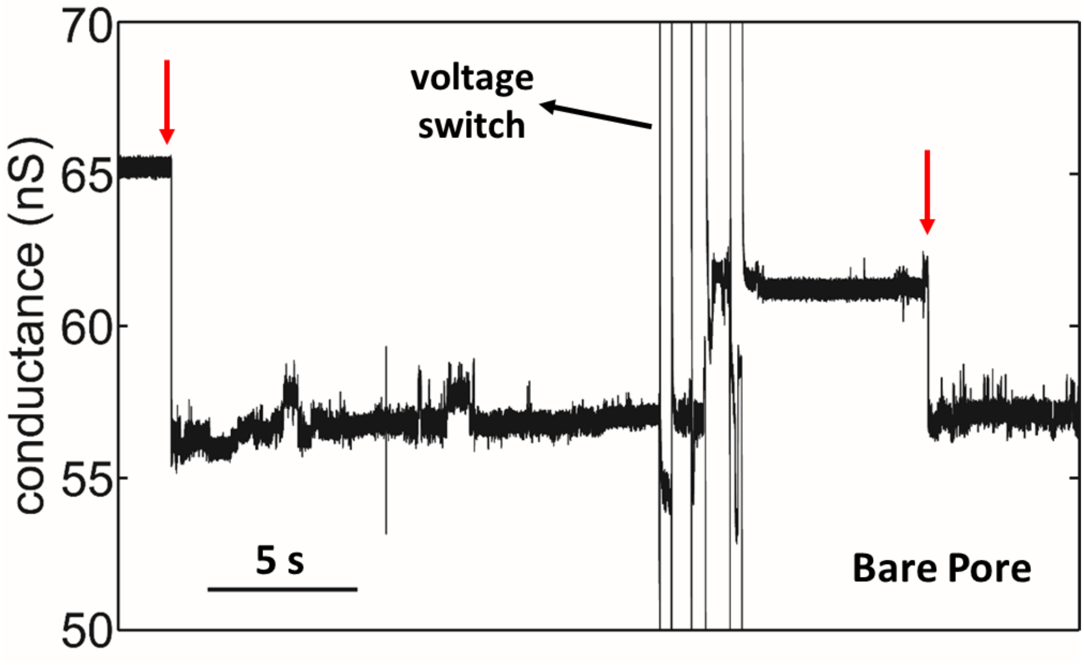

Supplementary Figure 1: Typical current trace for α-Syn detection experiment using a bare nanopore. Representative traces is recorded using a 20 nm nanopore under 100 mV. The base line is easily blocked after addition of α-Syn sample by observing a dramatic current drop (marked by red arrow) and a subsequent fluctuating current trace and it can hardly be unclogged by voltage switch, indicating it is an irreversible adsorption of α-Syn inside the nanopore.

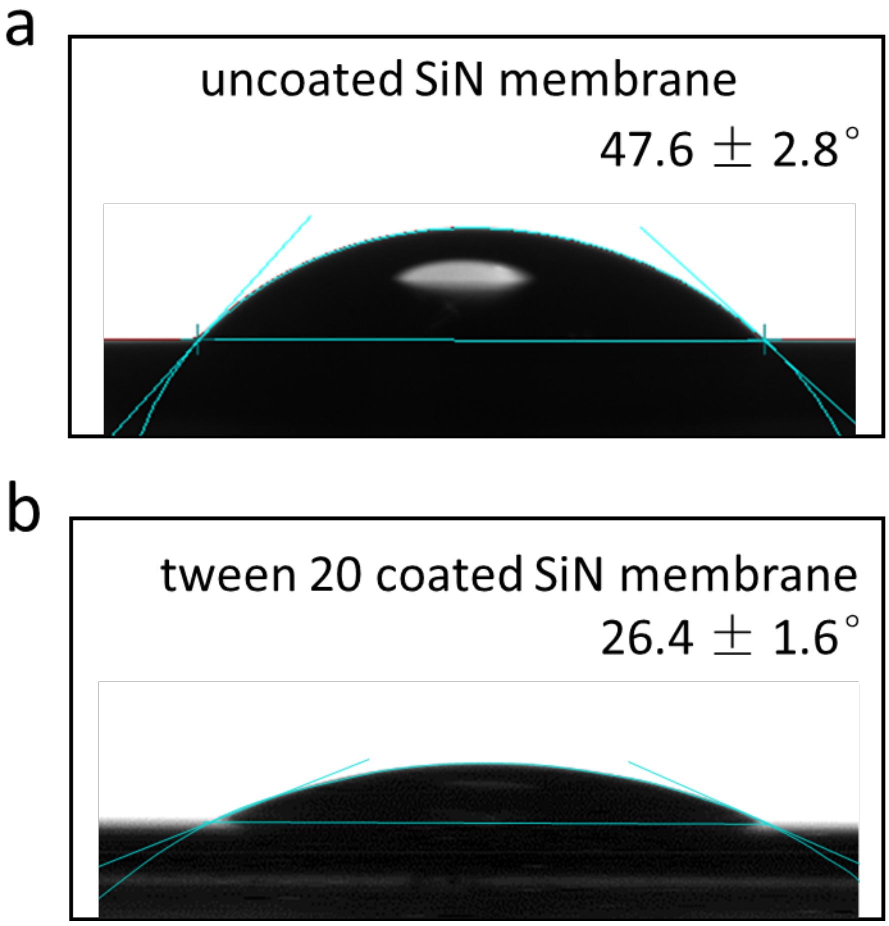

Supplementary Figure 2: Contact angle experiments for bare nanopore and tween 20 coated nanopore. Water contact angle between the liquid/solid interface and the liquid/vapor interface of **(a)** uncoated silicon nitride membrane and **(b)** tween 20 coated silicon nitride nanopore. The value of contact angle (CA) for bare nanopore is 47.6 ± 2.8° and is 26.4 ± 1.6° for tween 20 coated nanopore, indicating that tween 20 coating layer providing a more hydrophilic surface, which is believed to reduce the hydrophobic force between nanopore and α-Syn proteins and suppress non-specific adsorption of α-Syn[1, 2].

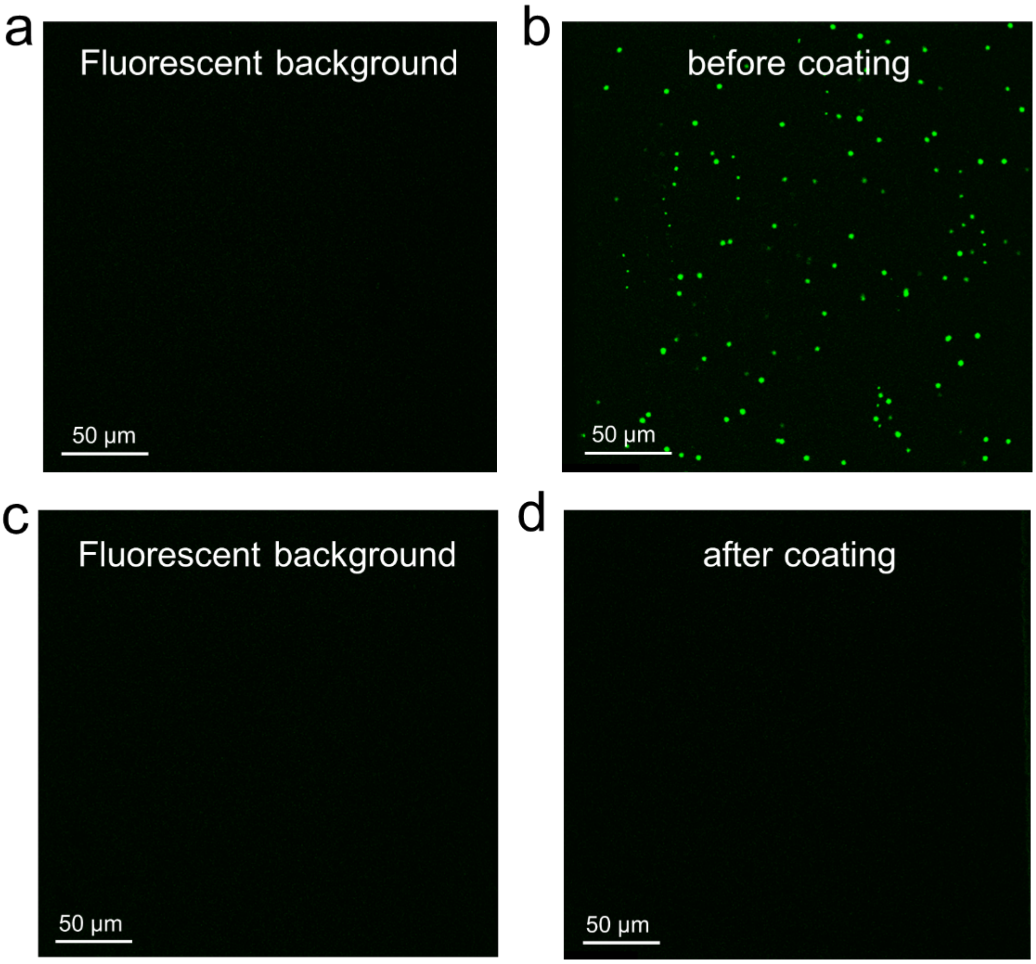

Supplementary Figure 3: The comparison of bare and Tween 20 coated cover glass surface in 1M KCl solution of pH 9 by fluorescent images. a). Fluorescent background before coating; b). 10 min after adding 48 h α-Syn sample; c). fluorescent background after coating; d). 10 min after adding 48 h α-Syn sample. The α-Syn sample was dyed by YOYO-1 for imaging with a confocal laser scanning microscope. The results are shown that dyed α-Syn oligomers are clearly seen on the uncoated surface, while almost no oligomers absorbed on Tween 20 coated surface, which provides a direct evidence that Tween 20 have an outstanding performance in inhibiting the adsorption of α-Syn oligomers.

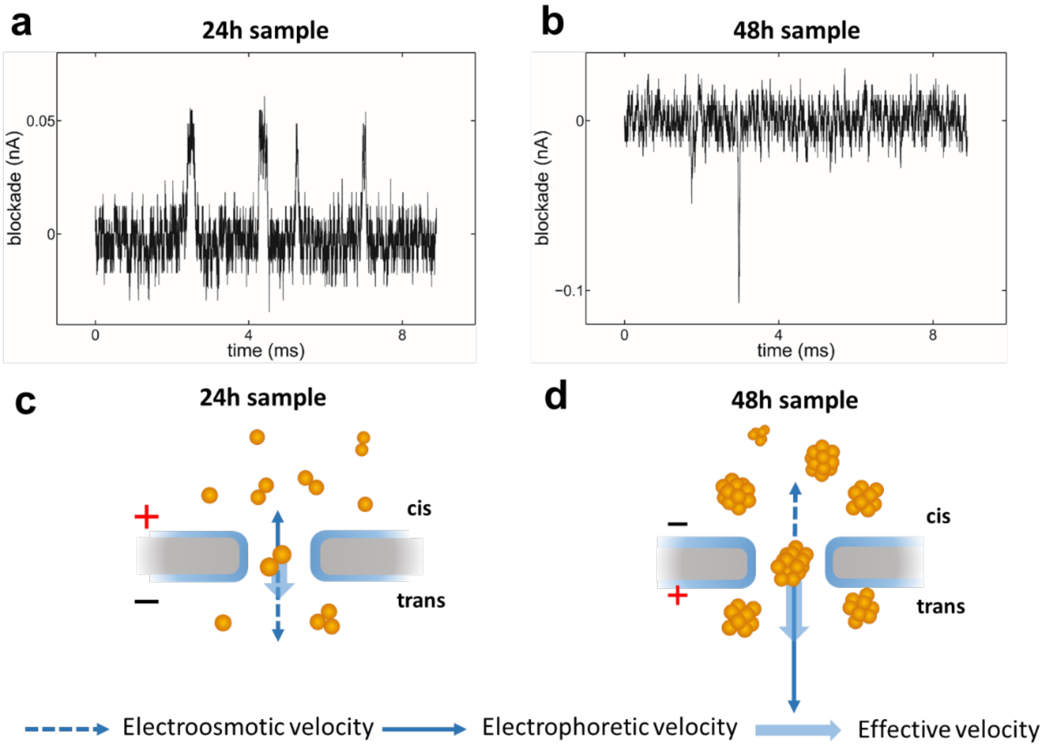

Supplementary Figure 4: Effect of electroosmosis in α-Syn only detection experiments. Typical traces for α-Syn samples incubated for **(a)** 24 h with V = -100 mV (electroosmosis dominant translocation) and **(b)** 48 h with V = 100 mV (electrophoresis dominant translocation) both in 1 M KCl at pH 9. We attribute this phenomenon to the competitive action of electrophoresis and electroosmosis, as illustrated in **(c)** and **(d)**. The effective velocity of the analyte can be written as $v_{eff} = \frac{\varepsilon E}{\eta}(\zeta_{protein} - \zeta_{pore})$[3]. In this equation, ε is permittivity; E is the electric field; η is the solution viscosity; ζ_{pore} is nanopore wall's zeta potential, and $\zeta_{protein}$ is the protein's zeta potential which is related to its charge. In our experiment condition, only $\zeta_{protein}$ changes along with incubation time. Due to addition of monomers, the charge density of aggregates become larger with longer incubation time, resulting in larger $\zeta_{protein}$ value. And $\zeta_{protein}$ finally exceeds ζ_{pore}, leading to the change of electroosmosis dominant translocation to electrophoresis dominant translocation from 24 h sample to 48 h sample.

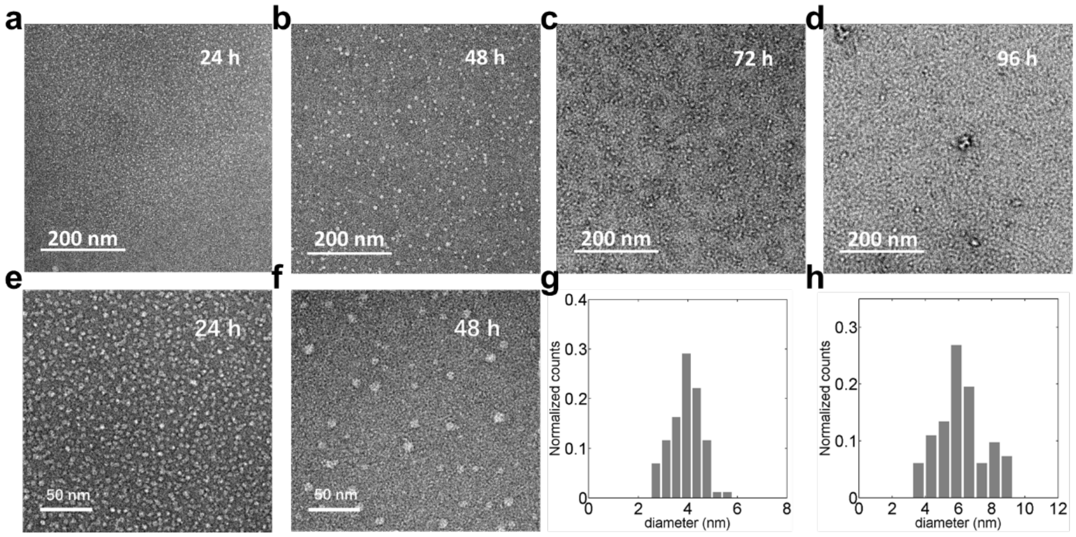

Supplementary Figure 5: Negative stain TEM images at 2.95k magnification for **(a)** 24 h sample, **(b)** 48 h sample, **(c)** 72 h sample, and **(d)** 96 h sample under controlled condition. Sample incubated for 24 h contains almost of oligomers with size around 4.6 nm. Oligomers with larger size emerge in samples incubated for 48 h, 72 h, and 96 h and the size of the α-Syn aggregates for these samples show heterogeneous distribution. The negative stain TEM image at 75k magnification of 24 h sample (e) and 48 h samples (f). Size analysis of 24 h sample (101 particles) (g) and 48 h (79 particles) samples (h).

It clearly shows the uniform size distribution of oligomer in 24 h sample with the size peak value of 4.0±0.1 nm, which is close to the value based on our nanopore experiment 4.4±0.1 nm. In the 48 h sample, the size of oligomers ranging from 4 nm to 9 nm based on TEM analysis, and the results from nanopore experiment ranges from 4.3 nm to 5.6 nm. The size of oligomers seems larger than the size determined by nanopore experiment may be due to the conformational change of large oligomers when adsorbed on the surface.

For the 72 h and 96 h samples, there are two difficulties in characterization with TEM. Firstly, the stained α-Syn oligomers in 96 h samples are hardly seen due to the decrease in concentration after aggregation, which makes it difficult to do statistical analysis; secondly, the magnified image of the 72 h and 96 h sample is not clear

enough due to a quite low contrast. As a result, it is hard to resolve the real size of oligomers in 72 h and 96 h sample. We attribute this to the complex formation process and structure of oligomers. The complex structure of oligomers makes the edge of oligomers blurring, making it really challenging to do quantitative single particle analysis.

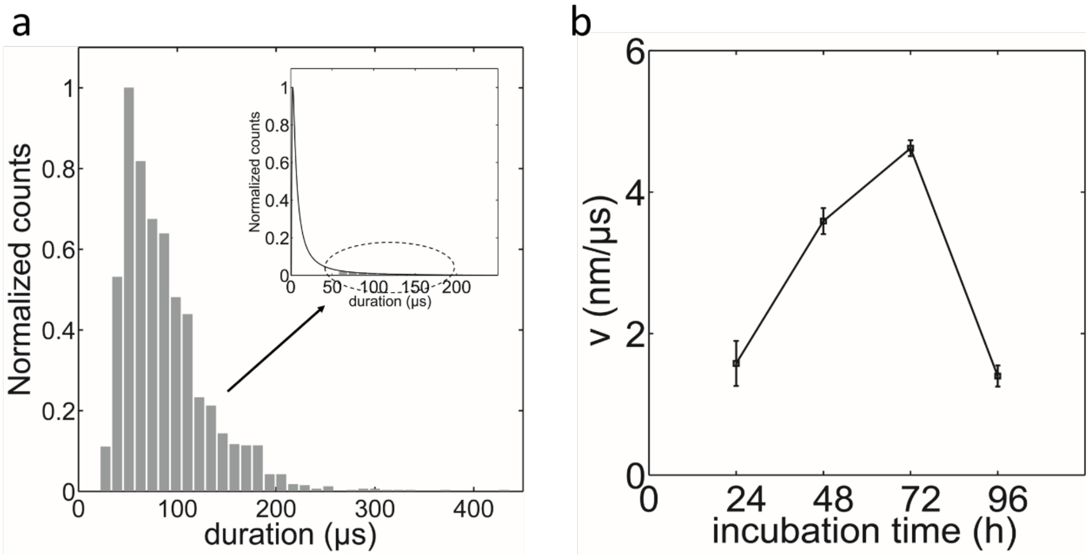

Supplementary Figure 6: **(a)** The event duration histogram for 24h α-Syn only sample and the inset is the corresponding fpt fitting result. Due to the small size of the analyte and limited temporal resolution in our nanopore probing experiments, the peaks in the duration histograms are artificial[4, 5]. So we exclude the peak values and those small than peak values in duration histograms and fit these by fpt formula reported by Carson et al. [6], with one parameter v. The distributions are normalized to their maximum value for clarity. **(b)** Extracted drift velocities from fpt fitting results for samples incubated at different time. The diffusion coefficient D is simply deduced through Stokes-Einstein relation, and the values are 111.6±2.5 $nm^2/\mu s$, 98.6±2.2 $nm^2/\mu s$, 73.0±3.0 $nm^2/\mu s$, and 88.2±2.4 $nm^2/\mu s$ for 24 h, 48 h, 72 h, and 96 h samples, respectively. The values of D decrease with longer incubation time up to 72 h is due to larger size of aggregates under incubation; the values of ν increase with longer incubation time up to 72 h is probably attributed to the increasing charge density of aggregates for monomers addition. The behavior for 96 h sample is maybe due to the increasing interaction between the relatively large aggregates and the inner wall of nanopore.

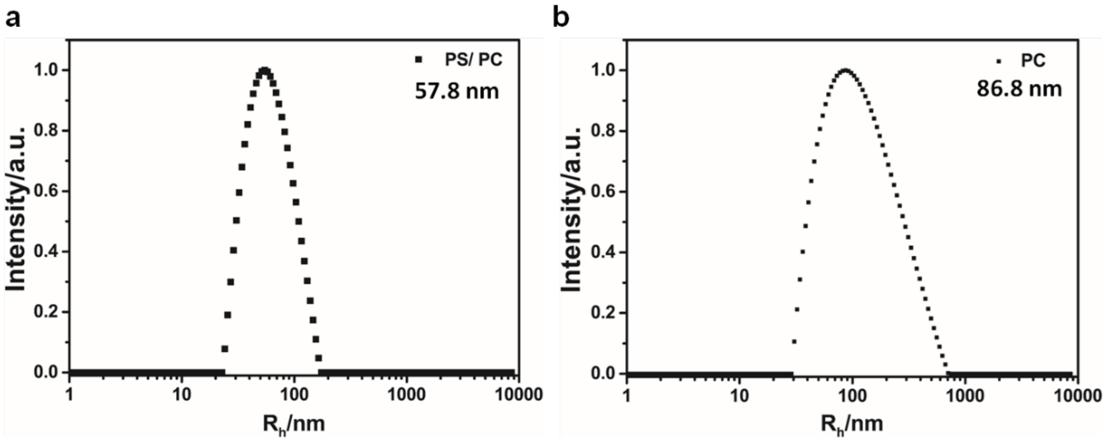

Supplementary Figure 7: Dynamic light scattering results for PS/PC & PC SUVs samples. The dynamic light scattering measurement shows that the size of composite phospholipid PS/PC SUVs is (a) 57.8 nm, is (b) 86.8 nm for the single component PC SUVs sample.

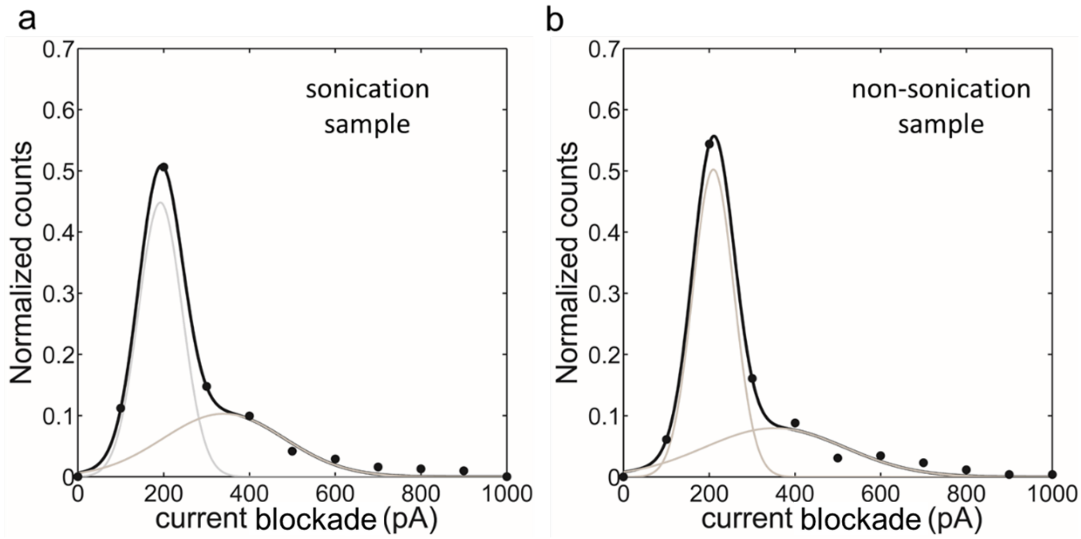

Supplementary Figure 8: The effect of ultrasonic treatment on incubated α-Syn only sample. α-Syn only samples incubated for two days with and without 30 s ultrasonic treatment tested. The Multi-Gaussian fitted current blockage histograms for the translocation events from **(a)** sonication sample and **(b)** non-sonication sample. Two Gaussian components are required to give flat residuals. The peak values are 192 ± 10.1 pA, 340.1 ± 106.6 pA and 209.6 ± 12.7 pA, 352.4 ± 130.6 pA for these two samples, respectively. We concluded that the sonication had limited effect on the structure on α-Syn aggregates based on almost the same current blockage distributions. Note that the current blockage values are generally much larger than that in our other experiments is because we used a thinner SiN membrane (50 nm) here[7].

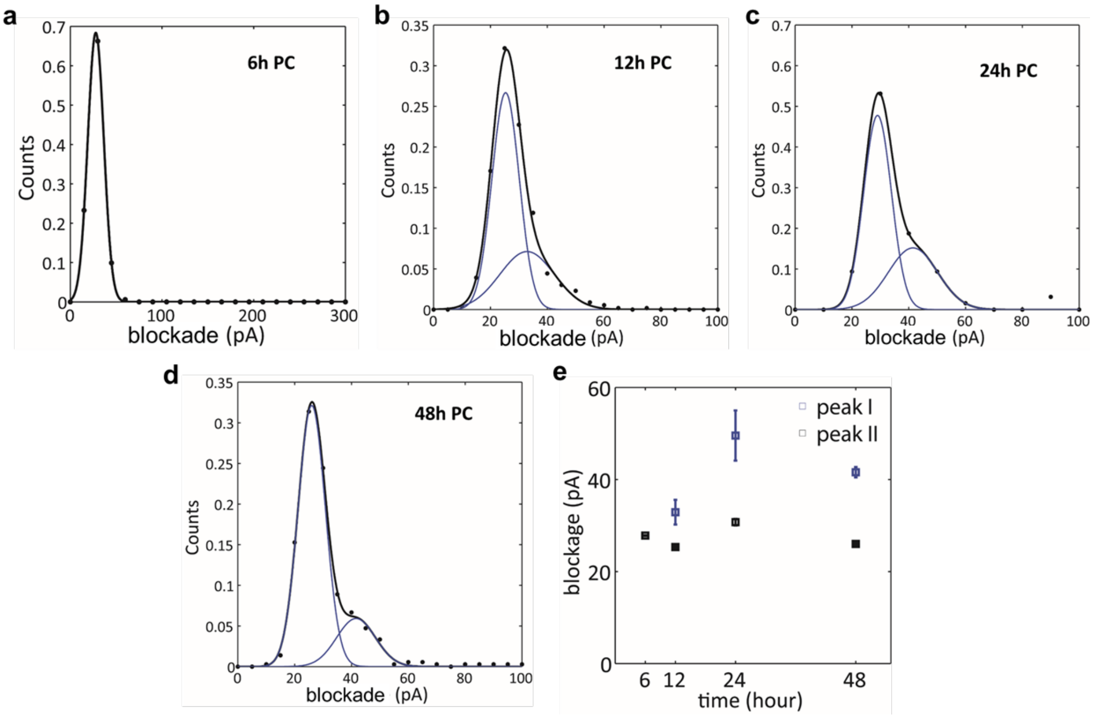

Supplementary Figure 9: Current blockage histograms for PC SUVs co-incubated α-Syn samples. Current blockage histograms for PC SUVs co-incubated samples for **(a)** 6 h, **(b)** 12 h, **(c)** 24 h, and **(d)** 48 h and corresponding multi-Gaussian fitting curves. **(e)** Peak values of Gaussian components for samples incubated at different time. The error arises from fitting error. No peak value exceeds 60 pA after 48 h of incubation indicating α-Syn, in the presence of only PC SUVs, indicating that PC has little effect on α-Syn aggregation.

Supplementary Table 1: The multi-Gaussian fitted peak position ΔI, calculated excluded volume Λ, and calculated oligomer diameter θ in 12 h, 18 h, 24 h, and 48 h lipid SUVs co-incubated α-Syn samples. The errors are fitting errors. The oligomers are regarded as spheres to calculate their diameter. (*the calculated diameter of O_{IV} based on the sphere model may lead to an overestimation of the actual size, which are more likely have an asymmetric short rod-like structure due to the broad distribution of blockage of O_{IV}[8] and the TEM image in Supplementary Figure 5d.)

**Sample**	**12 h**			**18 h**			**units**
**cluster**	-	O_I	-	-	O_I	-	-
ΔI	21.0±0.2	35.8±1.0	-	27.9±0.1	35.8±1.0	-	pA
Λ	30.0±0.2	51.1±1.4	-	39.8±0.1	51.2±1.3	-	nm^3
θ	3.9±0.1	4.6±0.1	-	4.2±0.1	4.6±0.1	-	nm

**Sample**	**24 h**			**48 h**			**units**
**cluster**	O_I	O_{II}	O_{III}	O_{III}	O_{IV}	-	-
ΔI	29.7±0.4	45.3±1.3	62.9±9.2	56.3±0.3	83.8±1.7	114.4±12.7	pA
Λ	42.4±0.6	64.8±1.8	132.0±29.0	80.4±0.5	119.7±2.4	163.4±18.1	nm^3
θ	4.3±0.1	5.0±0.1	5.6±0.3	5.4±0.1	6.1±0.1*	6.8±0.3*	nm

Supplementary References

[1] Shen L, Guo A, Zhu X. Tween surfactants: Adsorption, self-organization, and protein resistance. *Surf Sci* **605**, 494-499 (2011).

[2] Wei R, Rant U. Hydrophobic Interactions Retard Proteins upon Translocation through Silicon Nitride Nanopores. *Biophys J* **106**, 213A-213A (2014).

[3] Firnkes M, Pedone D, Knezevic J, Döblinger M, Rant U. Electrically Facilitated Translocations of Proteins through Silicon Nitride Nanopores: Conjoint and Competitive Action of Diffusion, Electrophoresis, and Electroosmosis. *Nano Lett* **10**, 2162-2167 (2010).

[4] Plesa C, Kowalczyk SW, Zinsmeester R, Grosberg AY, Rabin Y, Dekker C. Fast translocation of proteins through solid state nanopores. *Nano Lett* **13**, 658-663 (2013).

[5] Larkin J, Henley RY, Muthukumar M, Rosenstein JK, Wanunu M. High-Bandwidth Protein Analysis Using Solid-State Nanopores. *Biophys J* **106**, 696-704 (2014).

[6] Carson S, Wilson J, Aksimentiev A, Wanunu M. Smooth DNA Transport through a Narrowed Pore Geometry. *Biophys J* **107**, 2381-2393 (2014).

[7] Wanunu M, Dadosh T, Ray V, Jin J, McReynolds L, Drndic M. Rapid electronic detection of probe-specific microRNAs using thin nanopore sensors. *Nat Nanotech* **5**, 807-814 (2010).

[8] Yusko EC, et al. Controlling protein translocation through nanopores with bio-inspired fluid walls. *Nat Nanotech* **6**, 253-260 (2011).
